# The Endophytic Bacteria *Bacillus velezensis* Lle-9, Isolated from *Lilium leucanthum*, Harbors Antifungal Activity and Plant Growth-Promoting Effects

**DOI:** 10.4014/jmb.1910.10021

**Published:** 2020-02-05

**Authors:** Mohammad Sayyar Khan, Junlian Gao, Xuqing Chen, Mingfang Zhang, Fengping Yang, Yunpeng Du, The Su Moe, Iqbal Munir, Jing Xue, Xiuhai Zhang

**Affiliations:** 1 Beijing Agro-Biotechnology Research Center, Beijing Academy of Agriculture and Forestry Sciences, Beijing 00097, P.R. China; 2 Genomics and Bioinformatics Division, Institute of Biotechnology and Genetic Engineering (IBGE), The University of Agriculture, Peshawar 5000 Khyber Pakhtunkhwa, Pakistan; 3 Pharmaceutical Research Laboratory, Biotechnology Research Department, Ministry of Education, Mandalay Division, Kyaukse 05151, Myanmar

**Keywords:** Endophytic bacteria, antifungal activity, secondary metabolites, plant growth promotion

## Abstract

*Bacillus velezensis* is an important plant growth-promoting rhizobacterium with immense potential in agriculture development. In the present study, *Bacillus velezensis* Lle-9 was isolated from the bulbs of *Lilium leucanthum*. The isolated strain showed antifungal activities against plant pathogens like *Botryosphaeria dothidea*, *Fusarium oxysporum*, *Botrytis cinerea* and *Fusarium fujikuroi*. The highest percentage of growth inhibition *i.e.*, 68.56±2.35% was observed against *Fusarium oxysporum* followed by 63.12 ± 2.83%, 61.67 ± 3.39% and 55.82 ± 2.76% against *Botrytis cinerea*, *Botryosphaeria dothidea*, and *Fusarium fujikuroi*, respectively. The ethyl acetate fraction revealed a number of bioactive compounds and several were identified as antimicrobial agents such as diketopiperazines, cyclo-peptides, linear peptides, latrunculin A, 5α-hydroxy-6-ketocholesterol, (R)-S-lactoylglutathione, triamterene, rubiadin, moxifloxacin, 9-hydroxy-5Z,7E,11Z,14Zeicosatetraenoic acid, D-erythro-C18-Sphingosine, citrinin, and 2- arachidonoyllysophosphatidylcholine. The presence of these antimicrobial compounds in the bacterial culture might have contributed to the antifungal activities of the isolated *B. velezensis* Lle- 9. The strain showed plant growth-promoting traits such as production of organic acids, ACC deaminase, indole-3-acetic acid (IAA), siderophores, and nitrogen fixation and phosphate solubilization. IAA production was accelerated with application of exogenous tryptophan concentrations in the medium. Further, the lily plants upon inoculation with Lle-9 exhibited improved vegetative growth, more flowering shoots and longer roots than control plants under greenhouse condition. The isolated *B. velezensis* strain Lle-9 possessed broad-spectrum antifungal activities and multiple plant growth-promoting traits and thus may play an important role in promoting sustainable agriculture. This strain could be developed and applied in field experiments in order to promote plant growth and control disease pathogens.

## Introduction

The plant-associated bacteria termed as ‘plant growth-promoting rhizobacteria (PGPRs)’ have long been considered as important players in growth promotion and disease resistance in plants [1]. These PGPRs utilize several mechanisms to exert direct or indirect beneficial effects on associated plants. Synthesis of plant growth hormones, phosphate solubilization, nitrogen fixation, siderophores and ACC deaminase production are some of the key mechanisms through which these PGPRs promote plant growth [2, 3]. The PGPR-mediated disease resistance is conferred through various mechanisms either by blocking the rhizopshere colonization of pathogens or through secretion of antimicrobial compounds that degrade the cell walls of pathogens [4, 5].

Among the isolated PGPRs, several species of the genus *Bacillus* have been characterized as producing a variety of compounds and metabolites with antimicrobial and plant growth-promoting effects [6, 7]. For example, species such as *Bacillus licheniformis*, *Bacillus amyloliquefaciens*, and *Bacillus subtilis* have been proved very effective in controlling fungal diseases [8]. Some commercially available strains of these species are used as biofertilizers and biocontrol agents [9, 10]. In this connection, *Bacillus velezensis* is an important member of the *Bacillus* genus, and was first described as a heterotypic synonym of *B. amyloliquefaciens* [11]. Later, several other *Bacillus* species were reclassified as *Bacillus velezensis* based on genomics and DNA hybridization matches [12]. Several strains of *B. velezensis* have been isolated and characterized for their plant growth promotion and production of antifungal metabolites [13, 9]. Due to these properties, some strains of *B. velezensis* have been utilized in agriculture as plant growth promoters and biological control agents [14, 15, 12].

Lily, belonging to family *Liliaceae*, has significant aesthetic, medicinal and edible value. The genus *Lilium* contains about 100 species and is native to Asia, Europe, and North America. China, with 55 species, is considered the world's center of diversity when it comes to wild *Lilium* [16]. Lily bulbs have a centuries-long history of extensive utilization as a food source and traditional medicine in China. They have been used to provide nourishment as well as health-promoting properties to treat bronchitis and pneumonia [17, 18]. In addition, various *Lilium* species can also withstand harsh environmental conditions like drought and biotic stresses. In this connection, the role of plant-associated rhizosphere and endophytic microbes is crucial as some of the microbial metabolites seem to be characteristics of the biotype [19]. It was reported that some bioactive natural products recovered from plants had close similarities with products produced by the associated endophytes [20]. To the best of our knowledge, no reports are available of endophytic bacteria isolation from the bulbs of *Lilium* species up to date.

In the current study, an endophytic bacterial strain of *Bacillus velezensis* was isolated and identified from the bulbs of *Lilium leucanthum*. The isolated strain was further evaluated for antifungal, secondary metabolites and plant growth-promoting effects.

## Materials and Methods

### Sample Sterilization and Endophyte Isolation

Bulbs of *Lilium leucanthum* were collected from the experimental fields of Beijing Agro-Biotechnology Research Center, Academy of Agriculture and Forestry Sciences, China in December 2018.

The bulbs’ preparation, sterilization and isolation of endophyte were carried out using a previously described method [21]. The bulbs were peeled off, and the inner portions were washed with tap water for 5 min followed by treatment with 70% (v ⁄ v) ethanol for 1 min. The samples were then immersed in 10% (concentration of active chlorine) NaClO solution for 20 min and washed with sterile distilled water three times. After surface sterilization, the bulb portions were cut aseptically into approximately 1 cm × 1 cm pieces and placed on LB agar media plates. The plates were incubated at 30°C ± 1°C for 2-3 days until bacterial growth started on the cut bulb portions. The individual bacterial colonies appeared on bulb portions were aseptically inoculated into fresh LB broth and incubated at 30°C ± 1°C until pure cultures were obtained by serial sub-culturing.

### Morphological and Molecular Identification of Endophytic Bacteria

The isolated bacterial strain Lle-9 was characterized using colony morphology, growth pattern, Gram staining and scanning electron microscopic (SEM) analysis. The Gram reaction was performed as previously described [22]. Cell morphology of the isolate was determined using an SU8010 Field-Emission Scanning Electron Microscope (SEM, Hitachi, Japan).

For molecular analysis, the endophytic strain Lle-9 was cultured in LB broth for 24 h, incubated at 30°C in a shaker with 220 rpm. The culture was then centrifuged at 4,000 ×*g* for 15 min. Supernatant was discarded and the cell pellet was used for genomic DNA extraction using the Bacterial Genomic DNA Isolation Kit (SolarBio, China) in accordance with the manufacturer’s protocols. The isolated endophyte was identified by the sequences of 16S ribosomal RNA (rRNA) genes. A sequence of about 1,500 bp was amplified from genomic DNA using primers P027F and 1378R specific for the 16S ribosomal RNA genes. A 25 μl PCR reaction contained 1 μl (0.5-10.0 ng) of template DNA, 0.2 μM of each primer, P027F (5’-GAGAGTTTGATCCTGGCTAG-3) and 1378R (5’-CGGTGT GTACSSGGCCCGGGAACG-3’), 200 μM of each dNTP, 10X buffer, 2 mM MgSO_4_, and 1 U High-Fidelity KOD Taq DNA Polymerase. The cycle parameters were as follows: initial denaturation at 94°C for 4 min; 30 cycles of denaturation for 30 sec at 94°C, annealing for 1 min at 63°C, and extension for 1 min at 68°C; and a final overall extension for 7 min at 68°C. The PCR product was purified using the QIAquick PCR Purification Kit (Qiagen, Germany) and was then sent to Beijing Biomed Gene Technology Co., Ltd. for sequencing. Sequences were BLAST searched against homologous bacterial 16S ribosomal RNA sequences using NCBI. The determined sequences were aligned using ClustalW, and phylogenetic trees were constructed based on Neighbor-Joining (NJ) and Maximum Likelihood (ML) algorithms using the MEGA 7 software [23]. The nucleotide sequence was then submitted to GenBank under accession number MN461530.1.

### Antifungal Activity

In vitro antifungal assay was conducted to test the antagonistic effects of the isolated endophytic strain Lle-9 against four strains of pathogenic fungi, including *Botryosphaeria dothidea*, *Fusarium oxysporum*, *Botrytis cinerea* and *Fusarium fujikuroi*. The antifungal bioassays were conducted based on the dual culture method as previously described [24]. Zones of inhibition were calculated using the formula: % of growth inhibition = [(C − T)/C] × 100, where, C is the radial growth of the test pathogen in the control plates (mm), and T is the radial growth of the test pathogen in the test plates (mm). The experiment was repeated thrice.

### Ethyl Acetate Extraction of Secondary Metabolites

Secondary metabolites of the Lle-9 strain extracted by solvent partition method. The strain was grown in LB broth at 30°C and 150 rpm shaking for 5-6 days. The broth cultures were then centrifuged at 10,000 ×*g*, 4°C for 10 min. The supernatant was filtered through a 0.2 μm syringe filter. An equal volume of the filtrate and ethyl acetate was taken into the separating funnel and shaken for the complete extraction. The solvent phase that contains secondary metabolites was separated from the aqueous phase and solvent was evaporated to dryness to yield the crude extracts. The crude extract, about 20 mg, was re-dissolved in 1 ml of 70 % methanol. Then, 500 μl of dissolved extract was filtered through a 0.2 μm syringe filter before ultra high-performance liquid chromatography LTQ XL linear ion trap mass spectrometry/mass spectrometry (UHPLC-LTQ-XL-IT-MS/MS) analysis.

### UHPLC-LTQ-XL-IT-MS/MS Analysis for Secondary Metabolite Profiling

UHPLC-LTQ-IT-MS/MS analysis was performed using the method partially adapted from Lee *et al*. [25]. The Thermo Fischer Scientific LTQ XL linear ion trap mass spectrometry consisted of an electrospray interface (Thermo Fischer Scientific, USA) coupled with a DIONEX UltiMate 3000 RS Pump, RS Auto Sampler, and RS Column Compartment (Dionex Corporation, USA) that were used for secondary metabolite profiling of the fungal extracts. Sample was separated on a Thermo Scientific Hypersil GOLD C18 column with 1.9 μm particle size. The mobile phase consisted of A (0.1% (*v/v*) formic acid in water) and B (0.1% (*v/v*) formic acid in acetonitrile and the gradient conditions were increased from 10% to 100% of solvent B. Scanning was set to start after 1 min to source. Solvent gradient time was set over 19 min, and re-equilibrated to the initial condition for 4 min by setting the divert valve to waste. The flow rate was set at 0.3 ml/min and the injection volume was 10 μl. Temperature of the column during measurement was maintained at 35°C. Ion trap was performed in positive and full-scan ion modes within a range of 150–1,000 *m/z*. The operating parameters were as follows: source voltage, ±5 kV; capillary voltage, 39 V; capillary temperature, 275°C; auxiliary gas flow rate 10−20 arbitrary units; sheath gas flow rate 40−50 arbitrary units; spray voltage 4.5 kV. Tandem MS (MS/MS) analysis was performed by scan-type turbo data-dependent scanning (DDS) under the same conditions used for MS scanning for the six most intense ions using the Nth order double play mode. MS data were acquired by Xcalibar software (Thermo Fischer Scientific, USA).

### Putative Identification of Secondary Metabolites

Putative identification of secondary metabolites was done using molecular networking workflow from the GNPS website (https://gnps.ucsd.edu ) [26]. Raw LC-MS files were converted into mzXML using ProteoWizard 3.0.19140 [27], and the mzXML file was uploaded to GNPS. A molecular network was created using the default parameters. The spectra in the network were then searched against GNPS' spectral libraries. The library spectra were filtered in the same manner as the input data. All matches kept between network spectra and library spectra were required to have a score above 0.7 and at least 6 matched peaks.

### Plant Growth-Promoting (PGP) Assays

For plant growth-promoting assays, the bacterial strain Lle-9 was cultured in 1 ml LB media at 30°C for 48 h with 200 rpm shaking. The culture was then centrifuged at 4,000 ×*g* for 10 min at room temperature. The supernatant was removed and the cell pellet was washed thrice with 1 ml 10 mM MgSO_4_. The pellet was then re-suspended in 650 μl of MgSO_4_ and was used for PGP assays.

### ACC Deaminase Detection

The isolated strain Lle-9 was assayed for the production of 1-aminocyclopropane-1-carboxylate (ACC) deaminase according to the method previously developed by Belimov *et al*. [28] with minor modifications [29]. The color change from yellow to brown was considered as positive.

### Organic Acid Production Assay

Organic acids in the isolated strain Lle-9 were detected according to the protocol developed by Cunningham and Kuiack [30]. About 50 μl of the bacterial suspension in MgSO_4_ (10 mM) was inoculated in 800 μl of Sucrose Tryptone medium (ST) containing 20 g l^−1^ sucrose and 5 g l^−1^ tryptone. The ST medium was supplemented with 10 ml of trace element solution. Samples were incubated at 30°C and 200 rpm shaking for 5 days. After incubation, organic acids in samples were detected by adding 100 μl of 0.1% alizarine red S pH indicator. After 15 min, samples with yellow color were considered as positive. Pink-colored samples indicated negative results.

### Indole Acetic Acid (IAA) Detection

Indole acetic acid (IAA) production in the isolated strain Lle-9 was assayed according to the method of Gordon and Weber [31]. A bacterial suspension of 150 μl prepared in MgSO_4_ (10 mM) was inoculated in 3 ml of 1/10 diluted 869-rich medium. The medium was supplemented with various tryptophan concentrations of 0 mg ml^-1^, 2mg ml^-1^, 4 mg ml^-1^, and 6 mg ml^-1^. Samples were incubated at 30°C for 4 days with 150-180 rpm shaking in the dark. After incubation, the cultures were centrifuged at 4,000 ×*g* for 20 min. About 1 ml of the supernatant was mixed with 2 ml of Salkowski’s reagent (98 ml 35% HClO_4_, 2 ml 0.5M FeCl_3_). After 20 min, change of color from yellow to pink was considered as positive for IAA production. Quantitative measurement of indole acetic acid in the samples was conducted by measuring OD at 530 nm in a spectrophotometer. The IAA quantities in samples were measured based on a standard curve of known values (Fig. S1).

### Siderophore Detection

The siderophore production in the strain Lle-9 was evaluated through both qualitative and quantitative tests. The bacterial cells were cultured in liquid 284 medium with chrome azurol S (CAS) shuttle solution, according to the described method [32]. About 50 μl of the bacterial suspension in 10 mM MgSO_4_ was added to 800 μl of 284 medium prepared with three different iron concentrations. The iron concentrations used were: 0 μM, 0.25 μM, and 3 μM Fe(III) citrate. Samples were incubated at 30°C for 5 days with 150 rpm shaking. After incubation, 100 μl of the blue Chromium Azurol S (CAS) reagent was added to samples followed by incubation for 4 h at room temperature. After incubation, the change of color from blue to orange/yellow was considered as positive. Siderophore concentrations in all samples were further measured at 630 nm. The siderophore quantities were measured as % of siderophore units by the formula: % of siderophore units = Ar − As/Ar * 100 where, “Ar” is the absorbance of reference (CAS reagent); and “As” is the absorbance of sample at 630 nm. The ability of the strain to produce siderophores was further confirmed through qualitative test using CAS agar assay. All assays were carried out in triplicates.

### Nitrogen Fixation

Nitrogen fixing ability of the isolated strain Lle-9 was evaluated on nitrogen-deficient malate medium (NFM). *Escherichia coli* strain DH5α was used as negative control. A single colony of each endophytic strain Lle-9 and *E. coli* strain DH5α grown on solid LB medium were streaked onto solid nitrogen-deficient malate medium. Composition of this medium was: 0.1 g l^-1^ NaCl, 0.02 g l^-1^ CaCl_2_, 0.01 g l^-1^ FeCl_3_, 0.4 g l^-1^ KH_2_PO_4_, 0.5 g l^-1^ K_2_HPO_4_, 0.2 g l^-1^ MgSO_4_·7H_2_O, 0.002 g l^-1^ Na_2_MoO_4_·2H_2_O, 5 g l^-1^ sodium malate, 15 g l^-1^ agar, pH 7.2-7.4 using KOH) supplemented with 50 mg l^-1^ yeast extract [33]. Once colonies of the isolated strain appeared on NFM media, a resulting single colony was re-streaked onto NFM to confirm the ability to fix nitrogen [34]. Plates were incubated for 7 days at 28°C.

### Phosphate Solubilization

Phosphate solubilization was evaluated according to the method developed by Mehta and Nautiyal [35]. The *B. velezensis* strain Lle-9 was cultured on solid NBRIP medium (10 g l^-1^ glucose, 5 g l^-1^ Ca_3_(PO_4_)_2_, 5 g l^-1^ MgCl_2_, 0.1 g l^-1^ (NH_4_)_2_SO_4_, 0.25 g l^-1^ MgSO_4_·7H_2_O, 0.2 g l^-1^ KCl, 15 g l^-1^ agar). The ability of the strain Lle-9 to use inorganic phosphate in the form of Ca_3_(PO_4_)_2_ as a sole phosphate was determined. Plates were incubated for 14 days at 28°C.

### Experimental Design of Greenhouse Test

The growth-promoting effects of the isolated endophytic bacterial strain Lle-9 were evaluated on the Asiatic lilium hybrids ‘Tresor.’ Same-sized bulbs with normal and healthy appearance were selected from the storage house at 4°C. For inoculation, the isolated strain Lle-9 was cultured in 5 ml LB for 10-15 h followed by further inoculation in 50 ml LB for 24 h at 30°C with 220 rpm shaking. After incubation, the culture was re-inoculated in 400 ml LB and was kept to grow at 30°C for 24 h. This culture was then diluted 10 times with normal water and bulbs of Tresor variety were soaked in the diluted culture for 40 min. The non-inoculated bulbs, soaked in simple LB, were used as controls. Soil pots of sizes 20 × 30 cm were prepared with soil mix of peat moss, perlite, and vermiculite in a ratio of 2:1:1. Three lily bulbs, either inoculated or non-inoculated control were sown in each soil pot. Pots were kept in a completely randomized design (CRD). Each treatment contained 5 pots. Pots were kept in plastic trays with holes in the bottom. The plastic trays were watered with equal amounts of normal tap water at regular intervals. Morphological data such as number of flowering shoots, plant height, leaf length, leaf width, bulb size and weight and root length were taken at the peak vegetative and reproductive stage.

### Statistical Analysis

The data obtained were subjected to analysis of variance (ANOVA). Means were compared with Student’s *t*-test at a probability of α = 0.05.

## Results

### Isolation and Identification of *B. velezensis* Strain Lle-9

**Fig. 1:**
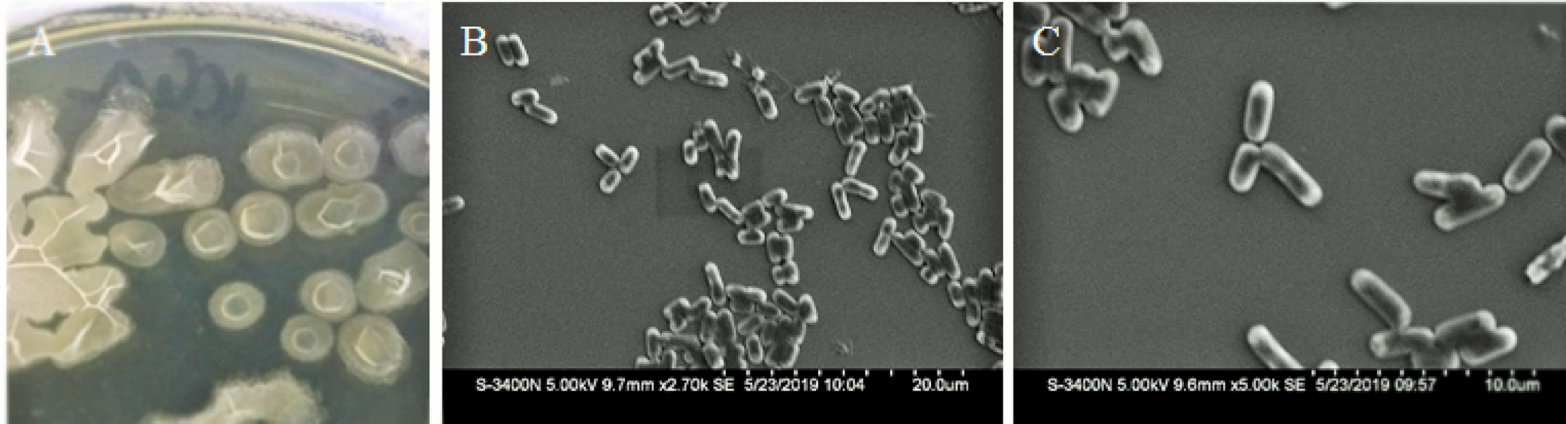
Colony morphology and scanning electron microscope (SEM) analysis of the endophytic bacterial strain of *B. velezensis* isolated from *L. leucanthum*. Strain Lle-9 produced whitish, chalky-colored colonies on LB agar plates (**A**). The isolate is of small rod-shaped structures (**B, C**).

In our study, several bacterial endophytes were isolated from the bulb samples of *Lilium leucanthum*. Based on the antifungal assays, the isolated endophytic strain Lle-9 of *B. velezensis* was selected for further assays. The isolated strain Lle-9 formed off whitish chalky colonies with internal whitish circular lines (Fig. 1A), as a gram-positive and spore-forming bacterium and exhibited small rod-shaped structures typical of the genus *Bacillus* as revealed by Scanning Electron Microscopic (SEM) analysis (Figs. 1B and 1C).

**Fig. 2:**
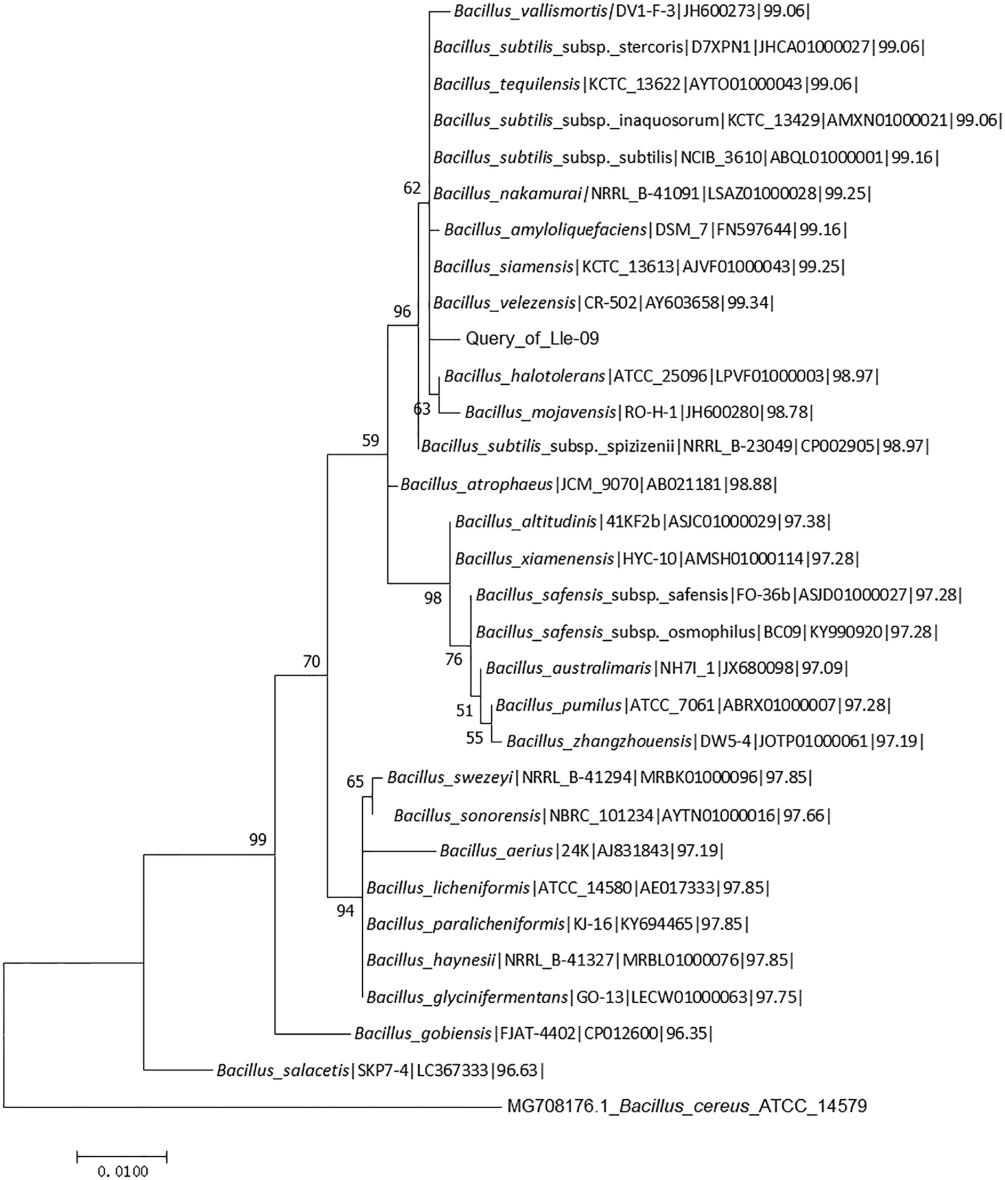
Phylogenetic analysis of 16S rRNA gene sequences of the bacterial endophyte Lle-9 isolated from *Lilium leucanthum*. Sequences were aligned through ClustalW using MEGA 7 software. Phylogenetic tree was constructed using Maximum Likelihood method. Bootstrap values are shown as percentages of 1000 replicates; values below 50% are not indicated. *Bacillus cereus* MG708176.1, ATCC14579 was used as an outgroup.

Molecular analysis indicated that the isolated strain Lle-9 belongs to the genus *Bacillus*. The BLAST results revealed that the 16S rRNA gene sequence was closely related to *Bacillus velezensis*. Based on the maximum likelihhod phylogenetic tree constructed with the 16S rRNA similarity (%), the Lle-9 strain revealed 99.34%similarity with *Bacillus velezensis* strain CR-502 (T), (AY603658) (Fig. 2). The 16S rRNA gene sequence of the isolated strain shared high similarities with other *Bacillus* species such as 99.25% with *Bacillus siamensis*, 99.25%with *Bacillus nakamurai*, and 99.16% with both *Bacillus amyloliquefaciens* and *Bacillus subtillus*. The 16S rRNA gene sequence of the isolated strain Lle-9 was submitted to GenBank under accession number MN461530.1.

### Antifungal Activity Analysis

**Fig. 3:**
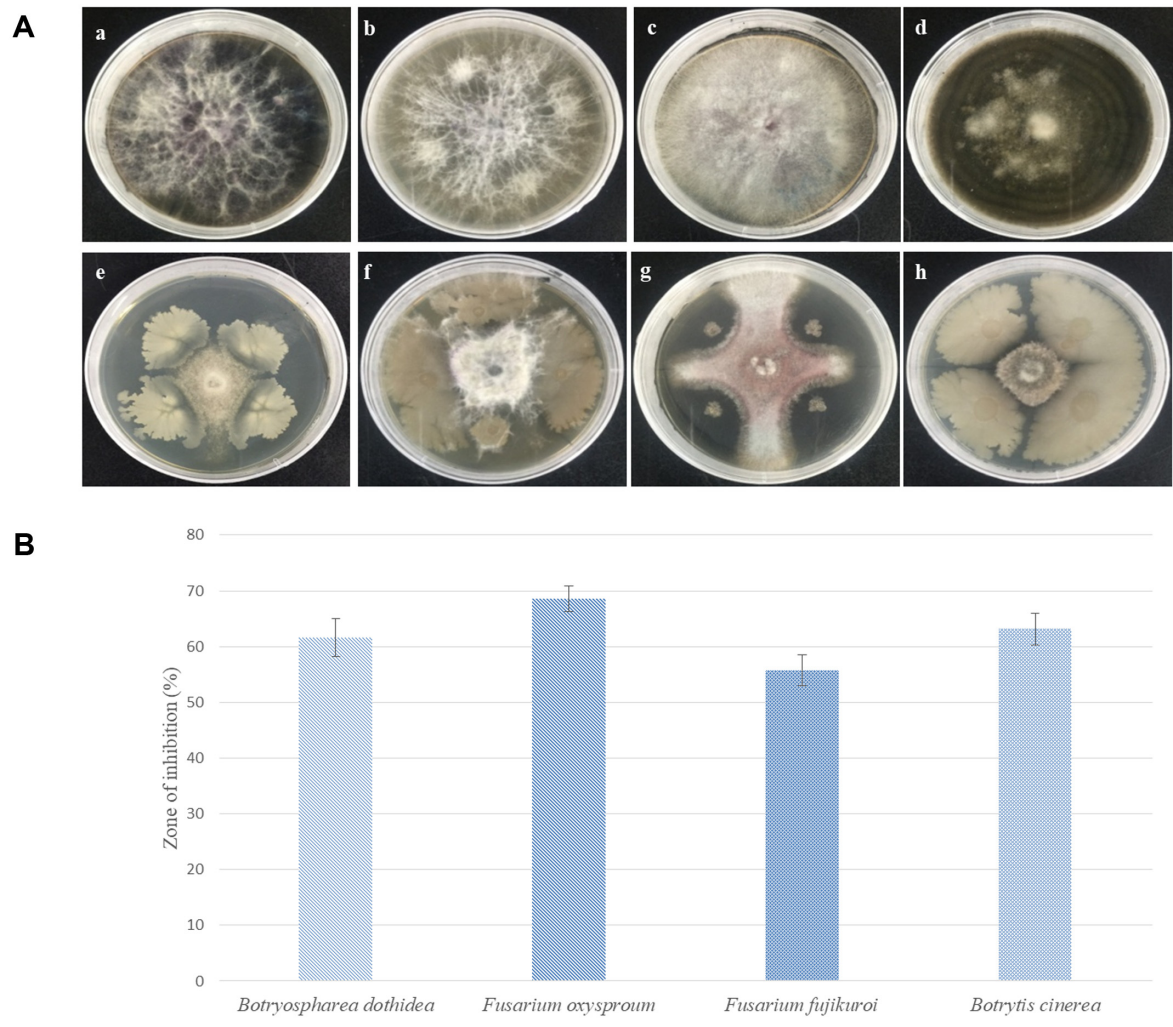
In vitro antifungal activities of the isolated endophytic bacterial strain Lle-9 from *Lilium leucanthum* against four fungal pathogens. (**A**) 5 mm fungus plug was inoculated into the center of PDA medium surrounded by four spots of bacterial inoculum. Plates (**A**), (**B**), (**C**), and (**D**) are controls of *Botryosphaeria dothidea*, *Fusarium oxysporum*, *Fusarium fujikuroi* and *Botrytis cinerea*, respectively. Plates (**E**), (**F**), (**G**), and (**H**) contain dual cultures of Lle-9 and the fungal pathogens. (**B**) Antifungal activities were measured as size of the zones of inhibition of the pathogenic fungi. Zones of inhibition were expressed as percentages.

The isolated endophytic strain Lle-9 showed high potential of broad-spectrum antifungal activities against the tested phytopathogens, *i.e.*, *Botryosphaeria dothidea*, *Fusarium oxysporum*, *Fusarium fujikuroi*, and *Botrytis cinerea* (Fig. 3A). These pathogenic strains were previously tested in an in vitro study for their pathogenicity potential and disease-causing ability in bulbs of Asiatic lilium hybrid ‘Tresor.’ All pathogenic fungal strains revealed disease symptoms in bulbs of Tresor (Fig. S2). The strain Lle-9 exhibited considerable inhibition potential as revealed by zones of inhibition of the test pathogens on PDA plates. These high activities might be due to the release of some diffusible compound(s) against the test pathogens. Zones of inhibition of pathogenic fungi on PDA plates were measured as percentage values. The highest percentage of growth inhibition, *i.e.*, 68.56 ± 2.35%, was observed against *Fusarium oxysporum* followed by 63.12 ± 2.83%, 61.67 ± 3.39%, and 55.82 ± 2.76%against *Botrytis cinerea*, *Botryosphaeria dothidea*, and *Fusarium fujikuroi*, respectively (Fig. 3B).

### Secondary Metabolite Analysis

LC/MS was used to characterize the potential bioactive secondary compounds in ethyl acetate fraction. The compounds identified putatively in the ethyl acetate fraction of *B. velezensis* Lle-9 are listed (Table S1). The compounds were characterized with molecular formula, m/z measured, library m/z, GNPS score, and GNPS library ID. A total of 34 putative compounds were identified by using molecular networking workflow from the GNPS website. Raw LC-MS files were converted into mzXML using ProteoWizard 3.0.19140, and the mzXML file was uploaded to GNPS. The spectra in the network were then searched against GNPS' spectral libraries. The compounds identified in the ethyl acetate fraction of *B. velezensis* Lle-9 were mostly diketopiperazines such as cyclo-dipeptides [cyclo(Ala-Leu), cyclo(D-Trp-L-Pro), cyclo-(Leu-Leu), and cyclo(Pro-Phe)], linear di-peptides [(Phe-Pro, Leu-Pro, Leu-His, Ile-Pro, Leu-Phe, Pro-Ile, Ile-Lys, His-Ile)], and tri-peptides [(Gln-Ile-Lys, and PyroGlu-Pro)]. In addition to these, some other bioactive compounds that were previously identified from various hosts as antifungal in nature were observed in the ethyl acetate fraction of Lle-9. The most important bioactive compounds were latrunculin A, 5alpha-hydroxy-6-ketocholesterol, (R)-S-lactoylglutathione, 1-(9Z-Octadecenoyl)-sn-glycero-3-phosphoethanolamine, triamterene, (2E,6E,10Z)-12-hydroxy-10-(hydroxymethyl)-6-methyl-2-(4-methylpent-3-enyl)dodeca-2,6,10-trienoic acid, 5S,12R,20-trihydroxy-6Z,8E,10E,14Z-eicosatetraenoic acid, rubiadin, moxifloxacin, 9-hydroxy-5Z,7E,11Z,14Z-eicosatetraenoic acid, D-erythro-C18-sphingosine, citrinin, 2-arachidonoyllysophosphatidylcholine, and (2R)-5,8-dihydroxy-2-(2-hydroxyphenyl)-7-methoxy-2,3-dihydrochromen-4-one.

### Plant Growth-Promoting (PGP) Assays

The plant growth-promoting effects of Lle-9 were assayed both qualitatively and quantitatively. According to our results, the endophytic bacterial strain Lle-9 showed positive results for all conducted assays.

### ACC (Deaminase) and Organic Acid Detection

**Fig. 4:**
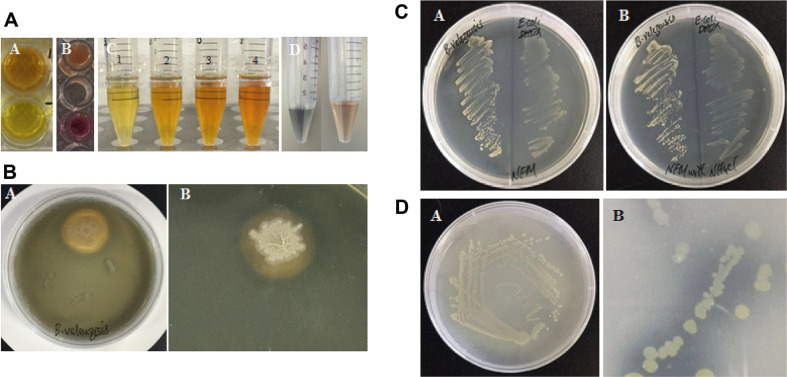
Qualitative detection of plant growth-promoting traits in the isolated strain. (**A**) ACC deaminase activity of Lle-9 (**A**). Lower well was used as negative control. Organic acid production in the strain Lle-9 (**B**). Extreme lower well with pink color was used as negative control. IAA detection in the strain showing change of coloration from yellow to pink (**C**). The tubes numbered with 1, 2, 3, and 4 show IAA detection at 0 mg ml^-1^, 2 mg ml^-1^, 4 mg ml^-1^, and 6 mg ml^-1^, respectively. Siderophore detection was confirmed by a change of color from blue to orange (**D**). (**B**) Siderophore detection on CAS blue agar plates. Siderophores were detected as a yellow/orange hallow surrounding the bacterial colonies (**A**). Closer look of the orange hallow surrounding the colony of Lle-9 (**B**). (**C**) Nitrogen-fixation by the endophytic strain Lle-9 of *B. velezensis*. The isolated strain was inoculated on nitrogen-deficient malate medium (NFM) and was assessed for growth in reference to non-nitrogenfixing *E. coli* DH5α. NFM medium (**A**); NFM supplemented with 5 mM NH_4_Cl (**B**). (**D**) Phosphate solubilization assay of *B. velezensis* le-09. Growth of Lle-9 on NBRIP medium (**A**). Closer look of the clearing area surrounding bacterial colonies (**B**).

The ACC deaminase production was detected through a qualitative test based on change of color. The strain Lle-9 was found positive for the production of ACC deaminase (Table 1 and Fig. 4A).

The isolated strain Lle-9 was assayed for production of organic acids through a qualitative test. Strain Lle-9 showed moderate to high production of organic acids as revealed by the change of color from pink to yellow (Table 1 and Figs. 4A and 4B).

### Indole Acetic Acid

Indole acetic acid (IAA) production in the isolated strain Lle-9 was detected through both qualitative and quantitative tests. Qualitative test confirmed IAA production in Lle-9 as revealed by the change of color of the culture supernatant from yellow to pink (Figs. 4A and 4C). Further, IAA was quantified in the strain at various tryptophan concentrations supplemented in the culture medium. The strain Lle-9 was able to produce IAA at different tryptophan concentrations (Table 1). Different tryptophan concentrations impacted the IAA production in the strain. The IAA content in the isolated strain increased with increasing tryptophan in the culture medium. The strain Lle-9 showed lower IAA content, *i.e.*, 23.2 ± 1.9 μg ml^-1^, at tryptophan concentration of 0 mg ml^-1^. However, the IAA content increased gradually with increasing the tryptophan concentrations from 0 mg ml^-1^ to 6mg ml^-1^ in the culture medium. The endophytic strain Lle-9 produced 79.7 ± 3.3 μg ml^-1^, 117.7 ± 3.5 μg ml^-1^, and 165.7 ± 5.8 μg ml^-1^ at tryptophan concentrations of 2 mg ml^-1^, 4 mg ml^-1^, and 6 mg ml^-1^ in the culture medium, respectively. These results suggest that the isolated strain Lle-9 has the potential to produce high content of indole acetic acid even in the absence of exogenous tryptophan. Application of exogenous tryptophan had no negative impact on IAA production; rather, it increased the production and a positive correlation was observed between the IAA production and the increasing tryptophan concentrations.

### Siderophores

Production of siderophores in Lle-9 was assayed both qualitatively and quantitatively at different Fe(III) citrate concentrations added to the culture medium. The endophytic strain Lle-9 was able to produce siderophores as confirmed through a change of color from blue to orange yellow (Figs. 4A and 4D). To further test whether an iron source has any impact on the production of siderophores, the strain was cultured in the liquid 284 medium supplemented with different Fe(III) citrate concentrations (Table 1). The strain Lle-9 showed high siderophore production when cultured in medium without Fe(III) citrate. The total siderophore quantity was reported as 51.3 ± 3.8 (psu) in the culture medium without addition of Fe(III) citrate. However, the siderophore accumulation by the strain declined as the quantities of Fe(III) citrate increased in the culture medium. Significantly greater decrease in siderophores was observed when Fe(III) citrate increased from 0 μM to 0.25 μM. About 33.3 ± 1.5 (psu) siderophores were detected at 0.25 μM Fe(III) citrate in the medium. Further reduction in siderophores was observed when Fe(III) citrate concentration was increased to 3.0 μM. However, this was not significantly different from the quantities obtained at 0.25 μM. At 3.0 μM Fe(III) citrate concentration, the strain Lle-9 accumulated 30.1 ± 1.3 (psu) siderophores. The siderophore production in the endophytic strain Lle-9 was further assayed through a qualitative test using chrome azurol S (CAS) on agar plates. An orange/yellow hallow was observed around the colonies of Lle-9 indicating the production of siderophores. The strain Lle-9 was able to quench the iron from the dye complex that resulted in a change of color from blue to orange/yellow in the form of a hallow surrounding the bacterial colony (Fig. 4B). Further, the diameter of the yellow/orange hallow produced by the strain averaged 15.32 ± 1.3 mm. This test further confirmed the high potential of the isolated endophytic strain Lle-9 to produce siderophores.

### Potential for Nitrogen Fixation and Phosphate Solubilization

The nitrogen-fixing potential of the isolated *B. velezensis* Lle-9 was assessed by its ability to grow on nitrogen-free minimal medium (NFM). The *Escherichia coli* strain DH5α, which is unable to grow on nitrogen-free medium was used as a negative control. Both strains were cultured on nitrogen-free medium and medium supplemented with 5 mM NH_4_Cl, which is a preferred source of nitrogen. Results revealed that the growth of *B. velezensis* Lle-9 was clearly visible on the NFM, while the *Escherichia coli* DH5α, which does not fix nitrogen, grew only on medium supplemented with reactive nitrogen (Fig. 4C). These results demonstrate the potential of the isolated endophytic *B. velezensis* Lle-9 to fix nitrogen.

The phosphate solubilization potential of the endophytic *B. velezensis* strain Lle-9 was assayed on solid NBRIP medium. In this medium, Ca_3_(PO_4_)_2_ was provided as the sole source of inorganic phosphate. The endophytic strain Lle-9 was able to grow on the medium for longer incubation time and it solubilized the inorganic phosphate as indicated by the clearing zone surrounding bacterial colonies (Fig. 4D).

### Plant Growth Promotion

**Fig. 5:**
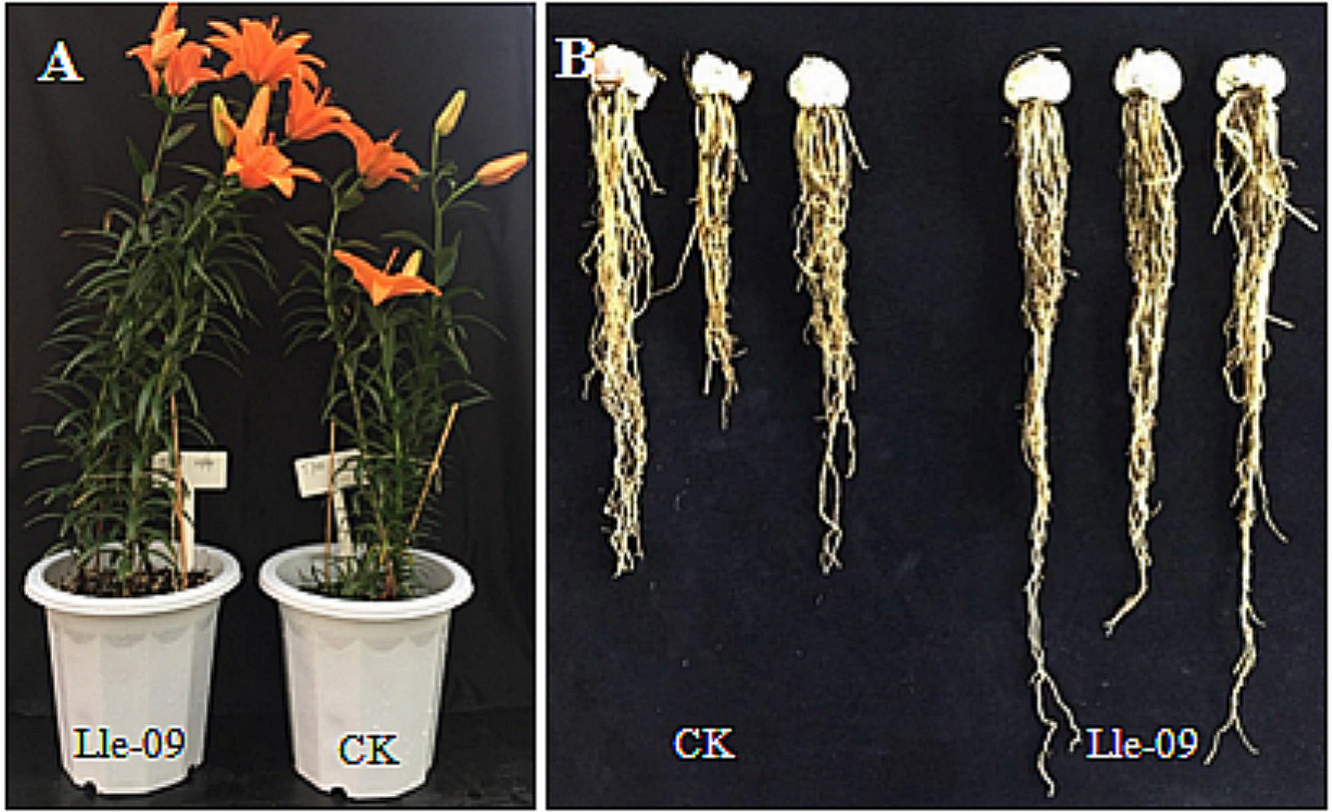
Plant growth promotion in Tresor variety upon inoculation of bulbs with the isolated strain Lle-9. Phenotypic differences of Lle-9 inoculated and un-inoculated (control) plants (**A**). Differences in bulbs and root elongation between CK and Lle-9 inoculated plants (**B**).

The plant growth-promoting potential of the isolated *B. velezensis* Lle-9 was further assessed on the vegetative growth and bulbs production of the Asiatic lilium hybrids ‘Tresor’ under greenhouse conditions. Bulbs of ‘Tresor’ were inoculated with the isolate strain before cultivation in soil pots. Upon completion of vegetative growth, a number of plant growth parameters like plant height, number of flowering shoots, leaf length, leaf width, stem diameter and weight of bulbs were measured between the inoculated and un-inoculated control plants. Our results revealed that inoculation of bulbs with *B. velezensis* Lle-9 resulted in significant increase in overall plant growth relative to control plants (Table 2). Inoculation of bulbs led to a significant increase in most of the tested growth parameters. The inoculated plants showed significantly high (*p* ≤ 0.05) increase in the number of flowering shoots, *i.e.* 3.47 ± 0.30, as compared to 2.73 ± 0.45 in un-inoculated control plants. Likewise, significant differences were observed in plant height between inoculated and un-inoculated plants (Fig. 5A, Table 2). Plants inoculated with Lle-9 showed plant height of 46.40 ± 4.98 cm as compared to 41.21 ± 4.69 cm of un-inoculated control plants. Leaf length also increased significantly in the inoculated plants relative to control plants. Inoculated plants showed significantly high (*p* ≤ 0.05) leaf length, *i.e.* 90.83 ± 8.96 mm, compared to 79.48 ± 11.83 mm in un-inoculated control plants. The inoculated plants showed improvement in leaf width relative to control plants; however, the differences were not significant. The *B. velezensis* Lle-9-inoculated plants showed 11.27 ± 1.24 mm average leaf width compared to 10.10 ± 1.24 mm of the control plants. Similar increase was observed in stem diameter in the inoculated plants relative to control plants. However, the differences were not significantly different. Below ground characteristics of the plants such as size and weight of bulbs and root elongation were also found different between the inoculated and non-inoculated control plants. Bulb sizes and weights were slightly improved in the inoculated plants compared to control plants; however, the differences were not significant. Inoculated plants developed significantly longer roots (26.34 ± 1.51 cm) as compared to non-inoculated control plants (19.23 ± 1.32 cm) (Fig. 5B). Overall, these results revealed that the *B. velezensis* Lle-9 was able to improve the vegetative and reproductive growth of lily plants upon inoculation.

## Discussion

In the present study, a new endophytic bacterial strain Lle-9 of *Bacillus velezensis* was isolated from the bulbs of *Lilium leucanthum*. The isolated strain was assessed for its potential of antifungal activities against several fungal pathogens and it showed antimicrobial activity and resisted the growth and proliferation of these pathogens. The strain was identified to be *B. velezensis* through morphological and molecular analysis. Several plant growth promotion assays were conducted to confirm whether the strain could promote growth of the associated plants through several underlined mechanisms and whether it could also provide protection against disease-causing agents. Previous studies indicated that several *B. velezensis* strains isolated from various hosts, showed broad-spectrum antimicrobial activities and plant growth promotion effects. *Bacillus amyloliquefaciens* FZB42, now recognized as a *B. velezensis* strain was reported in 2007 as the first gram-positive biocontrol bacteria to have its genome sequenced [36]. This isolate is now used as a model strain to promote plant growth and to confer disease resistance against broad-spectrum phytopathogens [37]. In addition, several other strains of *B. velezensis* have been used as antagonists of plant pathogens and as plant growth promoters in sustainable agriculture [12, 36, 38]. Further, a *Bacillus velezensis* strain CC09, isolated from healthy leaves of *Cinnamomum camphora*, showed immense potential as a new biocontrol agent, in control of many phytopathogenic diseases including wheat powdery mildew disease [39]. A *B. velezensis* strain NJAU-Z9, isolated from pepper rhizosphere, showed growth promotion effects in pepper [40].

In the present study, the isolated strain Lle-9 showed considerable antagonistic effects against fungal phytopathogens like *Fusarium oxysporum*, *Botryosphaeria dothidea*, *Botrytis cinerea*, and *Fusarium fujikuroi*. These pathogens have previously been reported to cause serious diseases in several crop plants [41]. These phytopathogens may cause diseases in *Lilium* species as revealed by a test confirming pathogenicity potential against cultivated species/varieties like *Lilium davidii* and Tresor. One of these phytopathogens, *Fusarium fujikuroi*, was isolated and identified from in vitro bulbs of *Lilium wardii*, a *Lilium* species. This could be a disease-causing agent in Lilum wardii and other Lilum species. Interestingly, the isolated endophytic strain Lle-9 exhibited higher antifungal activities against all tested fungal pathogens and was found very effective against growth and proliferation of *Fusarium fujikuroi*.

The ability of the strain Lle-9 to suppress the growth of phytopathogens could be due to the presence of compounds and metabolites with antimicrobial properties. To provide evidence of this, the ethyl acetate fraction was assessed for potential secondary metabolites with bio-control properties. A number of secondary metabolites were identified and showed close homologies with the already known compounds with bio-control properties. Some of the prominent compounds and group of compounds, putatively identified from the isolated strain were diketopiperazines, cyclo-peptides, linear peptides, latrunculin A, 5alpha-hydroxy-6-ketocholesterol, (R)-S-lactoylglutathione, triamterene, rubiadin, moxifloxacin, 9-hydroxy-5Z,7E,11Z,14Z-eicosatetraenoic acid, D-erythro-C18-sphingosine, citrinin, and 2-arachidonoyllysophosphatidylcholine. Isolation of these secondary metabolites and compounds is evidence that the Lle-9 has a high potential of restricting the growth and proliferation of disease-causing fungal pathogens. Our results are supported by previous identification of these bio-control compounds and metabolites from other *Bacillus* species. Diketopiperazines and linear di- and tripeptides were previously recovered from cultures of endophytic bacterial isolates. Syed-Ab-Rahman *et al*. [42] found diketopiperazines in the cultures of *Bacillus amyloliquefaciens*, *Bacillus velezensis*, and *Acinetobacter* sp. They further reported that the presence of these bioactive compounds resulted in resistance to *phytophthora* infection and plant growth promotion. A number of other studies also reported isolation of diketopiperazines (cyclo di-peptides) that resulted into broad-spectrum antimicrobial activities [43, 44]. Cyclo (Leu-Leu) found in *Lactobacillus plantarum* AF1 isolated from kimchi was found with antifungal activity against *Aspergillus flavus* [45]. Cyclo (Pro-Phe) in *Bacillus amyloliquefaciens* Q-426 was found to have a significant antifungal activity [46]. In addition, dipeptides and tripeptides have also been proved effective in conferring fungal resistance [47]. In the present study, latrunculin A was detected in the Lle-9 extract. This compound was previously shown as having fungistatic, fungicidal and fungilytic effects on the budding yeast *Saccharomyces cerevisiae* [48]. Triamterene, found in the ethyl acetate fraction of Lle-9 was previously found in ayurvedic medicine, *Salmali niryasa*, from a medicinal plant, *Bombax ceiba* [49]. The salmali resin had shown strong antimicrobial and antioxidant activities. Rubiadin, a bioactive compound detected in the present study was isolated from the roots of *Morinda elliptica* L.(Rubiaceae) [50]. This compound demonstrated anti-HIV, cytotoxic and antimicrobial activities [51]. Recently, rubiadin (AQ1) and rubiadin 1-methyl ether (AQ2), two photosensitizing anthraquinones (AQs) isolated from *Heterophyllaea pustulata*, showed reduction in biofilms formation of *Candida tropicalis*, a common cause of fungal infections [52]. Moxifloxacin was another important bioactive compound detected in the present study. This compound was previously shown as having antimicrobial effects [53]. The presence of these bioactive compounds might be responsible for the significant antifungal activities of Lle-9 against the tested fungal pathogens.

Previous studies have revealed that the plant growth-promoting traits in isolated strains of *Bacillus* species were correlated with several mechanisms such as production of organic acids, siderophores, lowering plant ethylene levels by ACC deaminase production, synthesis of plant growth-regulating hormones like indole-acetic acid and cytokinins, nitrogen fixation and phosphate solubilization [54]. In the present study, we confirmed through various tests that the isolated strain Lle-9 possessed plant growth-promoting traits. The isolated strain Lle-9 was capable of producing organic acids as revealed by a qualitative test. Organic acids play an important role in plant growth promotion and defense against phytopathogens [55].

Moreover, production of 1-aminocyclopropane-1-carboxylate (ACC) deaminase is one of the important characteristics of plant growth-promoting microbes and endophytes. ACC deaminase cleaves ACC, the immediate precursor of the plant hormone ethylene, to produce α-ketobutyrate and ammonia [56]. Ethylene serves as an important signaling molecule in plants under biotic and abiotic stresses and results in plant growth inhibition [57]. Previous studies have reported that inoculation of plants with ACC deaminase-producing microbes decreased ethylene levels that resulted in decreased inhibition of plant growth under biotic and abiotic stresses [3, 58]. Previous studies showed that improvement of several crops, inoculated with *Bacillus* species, might partly be due to the production of ACC deaminase. The *B. velezensis* strain BACO_3_ was reported to produce ACC deaminase, indole acetic acid, and ammonia, resulting in improved plant growth in terms of high biomass of leaves and roots in radish [59]. *Bacillus* species isolated from seeds of commercial tomato varieties exhibited multiple plant growth-promoting traits such as production of ACC deaminase, IAA, siderophores, and potential of nitrogen fixation and phosphate solubilization [60]. One of the isolates, HYT-12-1, identified as *Bacillus subtilis*, showed the highest ACC deaminase activity, which resulted in growth enhancement of tomato seedlings under greenhouse conditions. Consistent with the mentioned reports, the isolated strain Lle-9 in the present study exhibited ACC deaminase activity that might partly contributed to the improvement of growth of lily varieties upon inoculation.

Indole acitic acid is one of the important auxins that directly support plant growth and productivity. The ability of PGPRs including *Bacillus* species is partly attributed to their potential of IAA production that directly promotes growth of associated plants. In this connection, the role of the tryptophan precursor of IAA is important as considerable amounts of IAA are produced in the presence of excess tryptophan [61]. In the present study, the exogenous tryptophan concentrations had no negative impact on IAA production in Lle-9 and a positive correlation was observed between the tryptophan concentrations and IAA production. It seems the isolated strain Lle-9 produced IAA in a tryptophan-dependent pathway. Our results are supported by previous reports where application of exogenous tryptophan enhanced the IAA production. The *Bacillus amyloliquefaciens* strain FZB42, now a *B. velezensis* strain, was reported with production of IAA and its capacity increased five-fold with application of exogenous tryptophan [62]. *Bacillus thuringiensis* and *Bacillus cereus* isolated from soil samples exhibited IAA production in the absence and presence of L-tryptophan [63]. It was reported that the IAA production in the isolates increased with increasing concentrations of exogenous tryptophan.

Iron is an essential element necessary for growth of plants and microorganisms. However, it is abundantly present in soil in the form of insoluble Fe^3+^ oxy-hydroxides. Plant-associated microbes reduce Fe^3+^ to Fe^2+^ with the help of ferrireductases or solubilize it with extracellular Fe^3+^ chelators called ‘siderophores’ [64]. These soluble Fe^3+^-siderophore complexes are then available to both plants and microbes. Species of the genus *Bacillus* were previously reported with production of siderophores. Chen *et al*. (2007) reported siderophore bacillibactin production in *B. amyloliquefaciens* FZB42, now *B. velezensis*, and described that the siderophore synthesis in the strain was catalyzed by nonribosomal peptide synthetases. They further reported that production of high concentrations of siderophore bacillibactin in the FZB42 strain inhibited the growth of phytopathogenic bacterial and fungal competitors by depriving them of essential iron ions. Some recent studies also reported production of siderophores in *Bacillus* species [65, 66]. Kesaulya *et al*. [65] reported siderophore production in the isolated *Bacillus* sp. from potato rhizospheric soil. They further reported that the isolated strain inhibited the pathogen causing banana wilt disease. These reports reveal that siderophore production by the plant-associated microbes not only helps plants acquire access to limited iron supply in the soil but also confers plants with a selective advantage over the pathogenic microbes by depriving them of essential iron that leads to disease resistance. In the present study, the high siderophore production in the isolated strain Lle-9 might partly be responsible for considerable antagonistic effects against the tested pathogenic strains. Quantitative evaluation of siderophores was conducted under different iron concentrations. Maximum siderophore production was observed in the absence of Fe(III) citrate in the medium. However, increasing iron concentrations in the medium resulted in reduction in siderophore accumulation. These results are supported by previous reports where an inverse relationship was observed between siderophore production and different iron concentrations in the medium [67, 68].

Nitrogen is an essential and vital element for the normal growth and developments of plants. Many of the isolated PGPRs including species of *Bacillus* were found with the ability to fix atmospheric nitrogen. Syed-Ab-Rahman *et al*. [42] reported isolation of bacterial species including *B. amyloliquefaciens* (UQ154) and *B. velezensis* (UQ156) from soils of Arabidopsis plants. The *Bacillus* species were found with nitrogen-fixing ability. Other *Bacillus* species, which were isolated from *Sophora Alopecuroides* root nodules and rice showed nitrogen-fixing potential [69, 70]. Likewise, in the present study, the isolated endophytic *B. velezensis* strain Lle-9 showed nitrogen fixation ability by growth on nitrogen-free medium. In addition, the strain was able to solubilize inorganic phosphate on a solid NBRIP medium supplemented with Ca_3_(PO_4_)_2_ as the sole source of phosphate. Phosphate solubilization is one of the most important plant growth-promoting traits associated with endophytic bacteria.

The different strains belonging to the genus *Bacillus* have been proved very effective as biocontrol agents and biofertilizers in sustainable agriculture [38, 71]. Utilization of these bacteria has been found effective in controlling pathogenic microbes, exerting beneficial effects on plant growth and facilitating nutrient accessibility and assimilation [72-74]. Having these beneficial effects, the isolated strain Lle-9 was assessed for growth-promoting effects on the Asiatic lilium hybrid ‘Tresor’ under greenhouse conditions. Inoculated plants exhibited growth improvement and significant increases were observed for several growth parameters between the inoculated and non-inoculated control plants. Inoculated plants not only showed improved plant growth and bulb production but also produced a high number of flowers per plant. The improved growth performance in the Asiatic lilium hybrids ‘Tresor’ upon inoculation of Lle-9 might be due to the growth-promoting effects as were evaluated through several qualitative and quantitative tests. Previous studies also reported similar growth improvement when plants were inoculated with the isolated strains of *Bacillus velezensis* [75, 40]. The isolated *B. velezensis* strain Lle-9 showed antagonistic effects against the broad-spectrum fungal pathogens. These enhanced antifungal activities might be due to the presence of a number of bioactive compounds, which have previously been recognized as antimicrobial in nature. Moreover, the strain Lle-9 exhibited several plant growth-promoting traits, which were reflected in the improved vegetative and reproductive growth of lily plants upon inoculation. Owing to these beneficial antifungal and plant growth-promoting properties, the *B. velezensis* strain Lle-9 may be a good choice to be utilized as a source of bio-fertilizer and bio-control agent in sustainable agriculture.

## Supplemental Materials



Supplementary data for this paper are available on-line only at http://jmb.or.kr.

## Figures and Tables

**Table 1:** Plant growth-promoting (PGP) traits of the endophytic strain Lle-9 of *B. velezensis*.

OA	ACC		IAA (µg mL^-1^) at different tryptophan concentrations			Siderophores (psu) at different Fe(III) citrate concentrations			NA	PS
		0 mg mL^-1^	2 mg mL^-1^	4 mg mL^-1^	6 mg mL^-1^	0 µM	0.25 µM	3.0 µM		
++	++	23.2±1.9	79.7±3.3	117.7±3.5	165.7±5.8	45.2±3.6	35.1±1.3	21.5±1.2	++	++

Results are means ± standard deviation of three independent experiments with each treatment measured three times. Abbreviations are as follows: Organic acids (OA), ACC deaminase (ACC), Indole acetic acid (IAA), nitrogenase activity (NA), and phosphate solubilization (PS). Evaluation of the positivity to the tests; negative (−) shows absence of activity, while (+) shows lower to the highest activity (+++).

**Table 2:** Growth improvement in the Asiatic hybrids ‘Tresor’ upon inoculation with isolated strain Lle-9.

Treatments	# of flowering shoots	Plant height (cm)	Leaf length (mm)	Leaf width (mm)	Stem diameter (mm)	Bulb weight (g)	Root length (cm)
CK	2.73±0.45	41.21±4.69	79.48±11.83	10.10±1.24	7.89±0.74	12.66±1.20	19.23±1.32
Lle-9	3.47±0.30^*^	46.40±4.98^*^	90.83±8.96^*^	11.27±1.24	8.05±0.70	14.45±0.91	26.34±1.51^*^

Values are averages ±SD (*n* = 15). Values with asterisk (^*^) represent significantly different based on Student’s *t*-test (*p* < 0.05).
