# Supplementary material for: The Endophytic Bacteria Bacillus velezensis Lle-9, Isolated from Lilium leucanthum, Harbors Antifungal Activity and Plant Growth-Promoting Effects
Source: J Microbiol Biotechnol. 2020 Feb 5;30(5):668–80. doi: 10.4014/jmb.1910.10021 (PMC9728359; doi:10.4014/jmb.1910.10021)
Supplement: Supplementary file 1 [file JMB-30-5-668-supple.pdf]

J. Microbiol. Biotechnol.
https://doi.org/10.4014/jmb.1910.10021

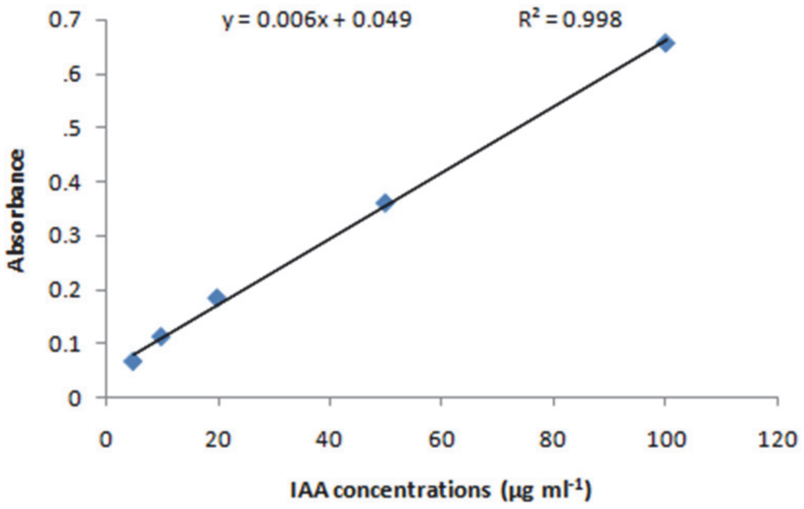

Fig. S1

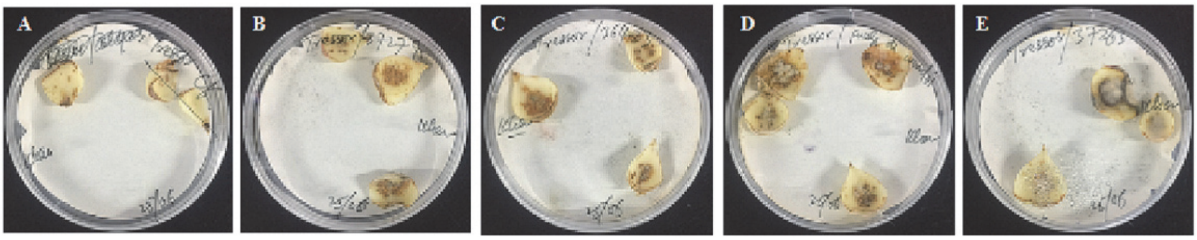

Fig. S2

Table S1: Overview of the putative compounds in the ethyl acetate fraction of *B. velezensis* Lle-9 strain.

No.	Compound name	m/z measured	Library m/z	Molecular fomula	adduct	GNPS score	GNPS Library ID	CAS no.
1.	cyclo(Ala-Leu)	185.034	185.128	$C_9H_{16}N_2O_2$	M+H	0.982933	CCMSLIB0000008 1144	
2.	His-Pro	235.114	235.12	$C_{11}H_{16}N_4O_3$	M+H-H2O	0.973503	CCMSLIB0000313 9663	N/A
3.	Phe-Pro	263.66	263.14	$C_{14}H_{18}N_2O_3$	M+H	0.963194	CCMSLIB0000313 9651	N/A
4.	Leu-Pro	212.024	211.144	$C_{11}H_{20}N_2O_3$	M+H-H2O	0.906355	CCMSLIB0000313 9607	N/A
5.	Leu-His	251.204	251.15	$C_{12}H_{20}N_4O_3$	M+H-H2O	0.880723	CCMSLIB0000313 8111	N/A
6.	Gln-Ile-Lys	389.436	388.251	$C_{17}H_{33}N_5O_5$	M+H	0.84873	CCMSLIB0000313 7672	N/A
7.	PyroGlu-Pro	228.775	227.103	$C_{10}H_{14}N_2O_4$	M+H	0.838431	CCMSLIB0000313 6001	N/A
8.	Latrunculin A	405.758	404.207	$C_{22}H_{31}NO_5S$	M+H-H2O	0.837671	CCMSLIB0000314 0065	76343936
9.	Ile-Pro	212.143	211.144	$C_{11}H_{20}N_2O_3$	M+H-H2O	0.828405	CCMSLIB0000313 9619	N/A
10.	5.alpha.-Hydroxy-6-ketocholesterol	418.411	419.355	$C_{27}H4_6O_3$	M+H	0.826932	CCMSLIB0000313 7907	13027333
11.	Leu-Phe	261.383	261.16	$C_{15}H_{22}N_2O_3$	M+H-H2O	0.822634	CCMSLIB0000313 6513	N/A
12.	(R)-S-Lactoylglutathione	379.134	380	$C_{13}H_{21}N_3O_8S$	[M+H]+	0.815957	CCMSLIB0000022 1231	25138-66-3
13.	1-(9Z-Octadecenoyl)-sn-glycero-3-phosphoethanolamine	503.8	502.29	$C_{23}H_{46}NO_7P$	M+Na	0.812591	CCMSLIB0000313 8664	89576294
14.	Triamtrene	252.685	254.115	$C_{12}H_{11}N_7$	[M+H]+	0.800298	CCMSLIB0000056 8753	N/A
15.	Dehydrocholic acid	383.449	385.237	$C_{24}H_{34}O_5$	M+H-H2O	0.793922	CCMSLIB0000313 7997	81232
16.	(2E,6E,10Z)-12-hydroxy-10-(hydroxymethyl)-6-methyl-2-(4-methylpent-3-enyl)dodeca-2,6,10-trienoic acid	358.624	359.219	$C_{20}H_{32}O_4$	[M+Na]+	0.790398	CCMSLIB0000470 9988	N/A
17.	5S,12R,20-Trihydroxy-6Z,8E,10E,14Z-eicosatetraenoic acid	209.969	211.096	$C_{20}H_{32}O_5$	211.1	0.775941	CCMSLIB0000313 6349	79516828
18.	cyclo(D-Trp-L-Pro)	284.499	284.139	$C_{16}H_{17}N_3O_2$	M+Na	0.775172	CCMSLIB0000000 7067	509078-49-3
19.	Cholesta-5,8(9)-dien-3.beta.-ol	383.453	385.343	$C_{27}H_{44}O$	M+H	0.77186	CCMSLIB0000313 7546	70741387
20.	Pro-Ile	228.904	229.16	$C_{11}H_{20}N_2O_3$	M+H	0.771714	CCMSLIB0000313 8037	N/A
21.	beta.-D-Allose	199.915	198.1	$C_6H_{12}O_6$	M+NH4	0.767589	CCMSLIB0000313 9104	7283092
22.	cyclo-(Leu-Leu)	227.194	227.174	$C_{12}H_{22}N_2O_2$	M+H	0.763656	CCMSLIB0000008 1186	
23.	Dehydrocholic acid	402.538	403.248	$C_{24}H_{34}O_5$	[M+H]	0.75731	CCMSLIB0000007 9041	N/A
24.	Rubiadin	256.102	255.065	$C_{15}H_{10}O_4$	M+H	0.750756	CCMSLIB0000084 7455	N/A

25.	Moxifloxacin	401.616	403.187	$C_{21}H_{24}FN_3O_4$	M+H	0.747011	CCMSLIB00000006194	N/A
26.	9-Hydroxy-5Z,7E,11Z,14Z-eicosatetraenoic acid	301.385	303.231	$C_{20}H_{32}O_3$	M+H-H_2O	0.744078	CCMSLIB00003137836	70968922
27.	Ile-Lys	260.914	260.2	$C_{12}H_{25}N_3O_3$	M+H	0.742579	CCMSLIB00003140024	N/A
28.	Estriol	270.235	271.2	$C_{18}H_{24}O_3$	[M-H_2O+H]+	0.741658	CCMSLIB00000213308	50-27-1
29.	Cyclo(Pro-Phe)	244.402	245.01	$C_{14}H_{16}N_2O_2$	M+H	0.739266	CCMSLIB00003134825	511126
30.	D-erythro-C18-Sphingosine	301.281	300.284	$C_{18}H_{37}NO_2$	M+H	0.731272	CCMSLIB00003139911	123784
31.	Citrinin	251.434	251.091	$C_{13}H_{14}O_5$	[M+H]+	0.724726	CCMSLIB00004720871	N/A
32.	His-Ile	253.029	251.15	$C_{12}H_{20}N_4O_3$	M+H-H_2O	0.717445	CCMSLIB00003138700	N/A
33.	2-arachidonoyllysophosphatidylcholine	542.461	543.333	$C_{28}H_{51}N_1O_7P_1$	M+H	0.706253	CCMSLIB00003130108	N/A
34.	(2R)-5,8-dihydroxy-2-(2-hydroxyphenyl)-7-methoxy-2,3-dihydrochromen-4-one	318.681	320.113	$C_{16}H1_4O_6$	[M+NH_4]+	0.703611	CCMSLIB00004700771	N/A
